# Michigan Marijuana Legalization: Correlations Among Cannabis Use, Mental Health, and Other Factors

**DOI:** 10.7759/cureus.27510

**Published:** 2022-07-31

**Authors:** Caleb Pawl, Angela Hong, Alyson McClintock, Chin-I Cheng, Juliette Perzhinsky

**Affiliations:** 1 Internal Medicine, Central Michigan University (CMU) College of Medicine, Mt. Pleasant, USA; 2 Statistics, Actuarial and Data Science, Central Michigan University, Mt. Pleasant, USA

**Keywords:** public health, prevalence, mental health, cannabis, marijuana legalization

## Abstract

Introduction: There are health implications with the statewide legalization of recreational marijuana that are still not fully understood and require further examination. This study evaluates the prevalence of marijuana use in patients being treated for a variety of conditions and whether correlations exist between marijuana use, mental health conditions, and concomitant use of psychotropic medications.

Methods: Data were collected from an electronic medical record (EMR) as part of a retrospective chart audit. A total of 500 charts were reviewed during a six-month timeframe from December 1, 2018 to May 31, 2019 with the start date approximating the timing of when marijuana became recreationally legalized in the State of Michigan.

Results: This study demonstrated a point prevalence of 15.8% since 79 of the 500 charts reviewed had marijuana use documented. Additionally, marijuana users were more likely to have a history of cocaine use, schizophrenia, antipsychotic use, and tobacco use.

Conclusion: Trends identified in this study provide a comparison point for the local prevalence of marijuana use immediately post state-wide legalization, with a projected increasing trend due to the removal of legal barriers.

## Introduction

In the United States (U.S.), the Controlled Substances Act of 1970 [1] prohibited the use of marijuana at the federal level. The controversy of marijuana legalization and its implications have gained traction politically and socially over the past decade. Although marijuana remains illegal at the federal level, there are currently 27 states that have decriminalized the possession of small quantities of marijuana, 36 states have legalized the medicinal use of marijuana, and 18 states within the US have legalized marijuana for recreational use [1]. During Michigan’s General Election on November 6, 2018, Ballot Proposal 18-1 was passed by a majority vote (56% in favor to 44% opposed) [1], making Michigan the first Midwest state to legalize recreational marijuana. When Proposal 18-1 went into effect on December 6, 2018, marijuana use became regulated similar to how alcohol sales are controlled. The public health impact of marijuana legalization remains controversial with unintentional intoxication risks in children, the combined use of marijuana with other drugs, driving safety concerns while using marijuana, and the unknown risk of other potential health problems such as pulmonary disorders or psychosis with chronic use of marijuana [2]. Supporters of legalization synthesize that there may be a decline in marijuana use in adolescents and a decrease in other illicit drugs by creating more robust policies and regulations for marijuana use and efficient use of law enforcement resources. With the lack of sufficient data available for the assurance of safety and efficacy of marijuana or standardization of dosing, professional medical associations like the American Society of Addiction Medicine, American Psychiatric Association, and American Medical Association oppose the legalization of marijuana [2]. Additionally, opposers are concerned legalization will create an increase in use and a potential increase in adverse health effects [2].

There are many implications of the statewide legalization of recreational marijuana that are still not fully understood and require additional investigation. Frequent cannabis use has been shown to increase after the legalization of cannabis [2]. In 2018, cannabis was the most commonly used substance worldwide with an estimated 192 million users globally [3]. Active marijuana use has been associated with problems including cognitive impairment, loss of motivation and coordination, memory and sleep disturbances, hyperemesis syndrome, psychological dependency, and mental health disorders such as schizophrenia, anxiety, and depression [4]. Heavier users of marijuana are shown to experience a first-episode psychosis [5,6], and an increasing number of patients being managed for psychosis/psychotic conditions are also reporting active marijuana use [7]. The long-term sequelae of these complications are not well studied, and as a result, may lead to increased disability and increased economic strain on healthcare systems [8]. Furthermore, previous literature indicates marijuana, specifically cannabidiol, may be useful for pain management [4,9], however, studies also revealed that the co-use of marijuana with opioids (also commonly used in treating pain) or alcohol has been shown to worsen overall health outcomes when compared to using opioids alone [10,11]. This is important to note since previous medical literature found a correlation in patients using marijuana with concomitant opioid use compared to non-opioid users [12,13].

Further implications include the correlation between active marijuana use and acute injuries, including fatal motor vehicle accidents (MVAs) [14,15] and workplace injuries [4]. The incidence of acute injuries secondary to MVAs should be examined as previous studies have shown that drug-impaired driving has become a major public safety concern in the US with marijuana as the most commonly detected drug other than alcohol in US drivers [14,15].

An additional concern with the legalization of marijuana is the normalization and increased social acceptance of marijuana use with increased legalization at a national level with increased distribution commercially. According to the Michigan Marijuana Regulatory Agency’s monthly report, in June 2020, there were 271 active licensed establishments in the State of Michigan for medical and recreational marijuana purposes, 123 of these include commercial retailers [16]. As marijuana continues to gain societal acceptance, its consideration as an “illicit drug” by the public becomes debatable, especially when used for medicinal purposes [17]. With some research showing the therapeutic benefits of marijuana, patients may not consider marijuana as a drug. Therefore, asking patients about their drug use raises concern about whether marijuana use is being monitored appropriately with unintentional or intentional reporter bias [18].

The primary aim of this study is to evaluate the prevalence of marijuana use among patients in the Mid-Michigan region that were evaluated in the emergency department (ED) or outpatient setting being treated for conditions associated with cannabis use, including mental health conditions and secondarily, acute injuries. The secondary aims of this study evaluated whether correlations exist between marijuana and acute injuries including work-related injuries and MVAs, co-usage with other pharmacological treatments, including opioids and psychotropic agents, and evaluate how the status of marijuana use is being documented by physicians and other health professionals.

This article was previously presented as a poster at the 2019 Michigan State Medical Society Foundation Annual Scientific Meeting on October 24, 2019.

## Materials and methods

Data was collected using a retrospective chart abstraction of 500 randomly selected adults (over the age of 18) seen in the Mid-Michigan region over a six-month timeframe between December 1, 2018 to May 31, 2019. Diagnoses were based on the 10th revision of the International Statistical Classification of Diseases and Related Health Problems (ICD-10) codes that have been correlated with active marijuana use such as anxiety, depression, schizophrenia, falls, cannabis use disorders, MVA, and acute injuries. Cannabis use disorders mentioned in this study include cannabinoid hyperemesis syndrome (F12.988), cannabis abuse (F12.10), cannabis dependence (F12.20), and cannabis intoxication (F12.92). Table 1 shows a comprehensive list of the ICD-10 diagnoses and their corresponding ICD-10 codes that were used as inclusion criteria for this study. Data were extracted using a standardized variable abstraction form of randomly selected charts meeting the ICD-10 inclusion criteria. This study was approved by the Hospital Institutional Review Board (IRB).

**Table 1:** ICD-10 diagnoses and corresponding diagnostic codes used as inclusion criteria for random selection of charts ICD-10: 10th revision of the International Statistical Classification of Diseases and Related Health Problems

ICD-10 Diagnoses	ICD-10 Code
Cannabis related disorders	F12
Motor- or Nonmotor-vehicle accident, type of vehicle unspecified	V89
Unspecified fall	W11
Injury unspecified	T14.90
Encounter for exam and observation following work accident	Z04.2
Acute pain due to trauma	G89.11
Mood [affective] disorders	F30-F39
Anxiety, dissociative, stress-related, somatoform, and other non-psychotic mental disorders	F40-48
Schizophrenia, schizotypal, delusional, and other non-mood psychotic disorders	F20-F29

Statistical analysis was done using SPSS Statistics version 26 (IBM Corp., Armonk, NY) [19]. To assess inter-rater reliability, Cohen’s Kappa was calculated in assessing the agreement between two raters for variables with binary outcomes, with a mean Cohen’s Kappa of 0.802, indicating substantial agreement and inter-rater reliability. Descriptive statistics were provided including mean (standard deviation) for body mass index (BMI) and count (percentage) for categorical variables. Confidence intervals for proportion were constructed based on a one-sample z-test. The two-sample t-test was used to test whether the average BMI is different between marijuana and non-marijuana users. The Chi-square test examined the association between marijuana use and sociodemographic variables including drug use. The Fisher’s exact test was the alternative to the Chi-square test when the cell count was small. All the analytical results were significant when p-values were less than or equal to 0.05.

## Results

A total of 79 encounters of the 500 (15.8%) charts reviewed had marijuana use documented. Sociodemographic factors were compared between marijuana users and non-marijuana users. It was found that 46.8% of marijuana users were female compared to 67.9% of non-marijuana users, while males had a higher prevalence of marijuana use at 53.2% (Table 1). It was also found that 62.0% of marijuana users were unemployed compared to 58.0% of non-marijuana users that were unemployed (Table 1). The relationship between marijuana and cocaine use was significant (p<0.001). Marijuana users were more likely to report cocaine use than were non-marijuana users with 10.1% of marijuana users reporting cocaine use vs. 0.5% of non-marijuana users (Table 2). The relationship between marijuana and tobacco use was also significant, (Chi2 (2)=44.81 [p<0.001]). Marijuana users were more likely to report previous or current tobacco use than were non-marijuana users (Table 2). The relation between marijuana use and schizophrenia was significant (p=0.018). Marijuana users were more likely to have schizophrenia than were non-marijuana users with 6.3% of marijuana users and 1.4% of non-marijuana users (Table 2). This study also found a higher prevalence of marijuana use in patients taking antipsychotics (19.0%) compared to non-marijuana users (10.0%) (Table 2), achieving statistical significance (Chi2 (1)=5.348 [p=0.021]).

**Table 2:** Descriptive statistics for variables in non-marijuana user and marijuana user strata Count (percentage) are listed. Exact=Fisher’s Exact test, Chi^2^=Chi-square test statistics. * : categories are combined for a valid Chi-square test.

Variable	Non-Marijuana User (n=421)	Marijuana User (n=79)	P-value	Test Statistics
Illicit Substance Use				
Cocaine use	2 (0.5%)	8 (10.1%)	<0.001	Exact
Rx Meds Abuse	4 (1.0%)	10 (12.7%)	<0.001	Exact
Other Sub use	4 (1.0%)	3 (3.8%)	0.083	Exact
Nicotine Status			<0.001	Chi^2^*(2)=44.81
Current Smoker	64 (15.2%)	34 (43.0%)		
Non-smoker	236 (56.1%)	16 (20.3%)		
Former Smoker	107 (25.4%)	23 (29.1%)		
E-Cigarettes	3 (0.7%)	3 (3.8%)		
Smokeless Tobacco	9 (2.1%)	3 (3.8%)		
Anxiety	273 (64.9%)	44 (55.7%)	0.120	Chi^2^(1)=2.40
Depression	169 (40.1%)	36 (45.6%)	0.370	Chi^2^(1)=0.81
Schizophrenia	6 (1.4%)	5 (6.3%)	0.018	Exact
Bipolar disorder	23 (5.5%)	6 (7.6%)	0.460	Chi^2^(1)=0.55
Unspecified injury	5 (1.2%)	0 (0.0%)	1.000	Exact
Motor-vehicle accident	13 (3.1%)	3 (3.8%)	0.727	Exact
Fall	42 (10.0%)	8 (10.1%)	0.970	Chi^2^(1)=0.00
Cannabis-related disorders	0 (0.0%)	11 (13.9%)	<0.001	Exact
Psyc Mgmt Antidepressants	253 (60.1%)	40 (50.6%)	0.120	Chi^2^(1)=2.46
Psyc Mgmt Antipsychotics	42 (10.0%)	15 (19.0%)	0.020	Chi^2^(1)=5.35
Psyc Mgmt Stimulants	14 (3.3%)	7 (8.9%)	0.034	Exact
Psyc Mgmt Mood Stabilizers	3 (0.7%)	1 (1.3%)	0.499	Exact
Psyc Mgmt Anxiolytics	157 (37.3%)	25 (31.7%)	0.720	Chi^2^(1)=1.35

Of the 500 patients reviewed, 62 had documented co-use of marijuana and other prescribed psychotropic(s), including antidepressants, antipsychotics, stimulants, mood stabilizers, and/or anxiolytics (see Table 3). Of the 62 with psychotropic and marijuana co-usage, 61.3% had a history of anxiety and 50% had a history of depression.

**Table 3:** Sociodemographic factors in non-marijuana users and marijuana users Mean (standard deviation) or count (percentage) are listed. Chi^2^=Chi-square test statistics, t=2 sample t-test statistics. *: categories are combined for valid Chi-square test. **: 95% Confidence Interval for proportion.

Variable	Non-Marijuana User (n=421)	Marijuana User (n=79)	P-value	Test Statistics
	n (%) / n (Mean (S.D.))	n (%) / n (Mean (S.D.))		
Prevalence		15.80%		
Sex			<0.001	Chi^2^11)=12.95
Female	286 (67.9%)	37 (46.8%)		
Male	135 (32.0%)	42 (53.2%)		
Ethnicity			0.106	Chi^2^*(1)=2.61
White	378 (90.0%)	66 (83.5%)		
Black	25 (6.0%)	10 (12.7%)		
Hispanic	9 (2.1%)	3 (3.8%)		
Asian	2 (0.5%)	0 (0.0%)		
Other	4 (1.0%)	0 (0.0%)		
Care Setting			<0.001	Chi^2^(1)=23.27
Outpatient	387 (91.9%)	58 (73.4%)		
ED	34 (8.1%)	21 (26.6%)		
BMI	403 (31.0 (7.7))	73 (29.4 (8.6))	0.110	t=1.61
Employment Status			<0.001	Chi^2^(1)=24.92
Unemployed	244 (58.0%)	49 (62.0%)		
Insurance Type			0.322	Chi^2^*(1)=1.01
Employee sponsored	195 (46.3%)	32 (40.5%)		
State sponsored	222 (52.7%)	47 (59.5%)		
None	1 (0.2%)	0 (0.0%)		
Using Marijuana to manage				
Anxiety		11 (13.9%) (6.29%, 21.56%)**		
Pain Management		14 (17.7%) (9.30%, 26.14%)**		

Of the 500 patients reviewed, 62 had documented co-use of marijuana and other prescribed psychotropic(s), including antidepressants, antipsychotics, stimulants, mood stabilizers, and/or anxiolytics (Table 4). Of the 62 with psychotropic and marijuana co-use, 61.3% had a history of anxiety and 50.0% had a history of depression.

**Table 4:** Descriptive statistics in co-usage for both marijuana and psychotropics Test statistics (p-value) are for comparison between marijuana only and co-usage. Mean (standard deviation) or count (percentage) are listed. Exact=Fisher’s Exact test, Chi^2^=Chi-square test statistics, t=2 sample t-test statistics. * : categories are combined for valid Chi-square test.

Variable	Neither (n=100)	Psychotropic only (n=321)	Marijuana only (n=17)	Co-usage (n=62)	P-value	Test Statistics (p-value)
Sex					0.598	Chi^2^(1)=0.28
Female	62 (62.0%)	224 (69.8%)	7 (41.2%)	30 (48.4%)		
Male	38 (38.0%)	97 (30.2%)	10 (58.8%)	32 (51.6%)		
Ethnicity					0.375	Chi^2^*(1)=0.79
White	85 (85.0%)	293 (91.6%)	13 (76.5%)	53 (85.5%)		
Black	9 (9.0%)	16 (5.0%)	2 (11.8%)	8 (12.9%)		
Hispanic	4 (4.0%)	5 (1.6%)	2 (11.8%)	1 (1.6%)		
Asian	0 (0.0%)	2 (0.6%)	0 (0.0%)	0 (0.0%)		
Other	0 (0.0%)	4 (1.3%)	0 (0.0%)	0 (0.0%)		
Care Setting					0.005	Chi^2^(1)=7.71
Outpatient	80 (80.0%)	307 (95.6%)	8 (47.1%)	50 (80.7%)		
ED	20 (20.0%)	14 (4.4%)	9 (52.9%)	12 (19.4%)		
BMI	88 (31.0 (7.5))	315 (31.0 (7.7))	12 (27.3 (4.6))	61 (29.8 (9.2))	0.169	t=-1.41
Employment Status					0.046	Chi^2^(1)=4.00
Employed	44 (44.0%)	133 (41.4%)	10 (58.8%)	20 (32.3%)		
Insurance Type					0.107	Chi^2^(1)=2.59
Employee Sponsored	44 (44.0%)	151 (47.0%)	4 (23.5%)	28 (45.2%)		
State Sponsored	54 (54.0%)	168 (52.3%)	13 (76.5%)	34 (54.8%)		
None	1 (1.0%)	0 (0.0%)	0 (0.0%)	0 (0.0%)		
Illicit Substance Use						
Active use	2 (2.0%)	1 (0.3%)	0 (0.0%)	5 (8.2%)	0.579	Exact
Former use	1 (1.0%)	7 (2.2%)	3 (17.7%)	12 (19.4%)	0.874	Chi^2^(1)=0.03
Cocaine Abuse	0 (0.0%)	2 (0.6%)	1 (5.9%)	7 (11.3%)	0.513	Chi^2^(1)=0.43
Heroin Abuse	0 (0.0%)	1 (0.3%)	0 (0.0%)	4 (6.5%)	0.572	Exact
Rx Meds Abuse	0 (0.0%)	4 (1.3%)	2 (11.8%)	8 (12.9%)	0.900	Chi^2^(1)=0.02
Other Sub Abuse	3 (3.0%)	1 (0.3%)	1 (5.9%)	2 (3.2%)	0.522	Exact
Nicotine Status					0.953	Chi^2^(1)=0.10
Current Smoker	16 (16.0%)	48 (15.0%)	9 (52.9%)	25 (40.3%)		
Non-smoker	61 (61.0%)	175 (54.5%)	3 (17.7%)	13 (21.0%)		
Former Smoker	21 (21.0%)	86 (26.8%)	5 (29.4%)	18 (29.0%)		
E-Cigarettes	0 (0.0%)	3 (0.9%)	0 (0.0%)	3 (4.8%)		
Smokeless Tobacco	2 (2.0%)	7 (2.2%)	0 (0.0%)	3 (4.8%)		
Anxiety	37 (37.0%)	236 (73.5%)	6 (35.3%)	38 (61.3%)	0.056	Chi^2^(1)=3.65
Depression	24 (24.0%)	145 (45.2%)	5 (29.4%)	31 (50.0%)	0.131	Chi^2^(1)=2.28
Schizophrenia	1 (1.0%)	5 (1.6%)	1 (5.9%)	4 (6.5%)	0.932	Chi^2^(1)=0.01
Bipolar disorder	4 (4.0%)	19 (5.9%)	0 (0.0%)	6 (9.7%)	0.331	Exact
Unspecified injury	2 (2.0%)	3 (0.9%)	0 (0.0%)	0 (0.0%)		---
Motor-vehicle accident	11 (11.0%)	2 (0.6%)	1 (5.9%)	2 (3.2%)	0.522	Exact
Work-related injury	0 (0.0%)	0 (0.0%)	0 (0.0%)	0 (0.0%)		---
Fall	31 (31.0%)	11 (3.4%)	4 (23.5%)	4 (6.5%)	0.061	Exact
Cannabis related disorders	0 (0.0%)	0 (0.0%)	4 (23.5%)	7 (11.3%)	0.238	Exact
Psyc Mgmt Antidepressants	0 (0.0%)	253 (78.8%)	0 (0.0%)	40 (64.5%)	<0.001	Exact
Psyc Mgmt Antipsychotics	0 (0.0%)	42 (13.1%)	0 (0.0%)	15 (24.2%)	0.032	Exact
Psyc Mgmt Stimulants	0 (0.0%)	14 (4.4%)	0 (0.0%)	7 (11.3%)	0.336	Exact
Psyc Mgmt Mood Stabilizers	0 (0.0%)	3 (0.9%)	0 (0.0%)	1 (1.6%)	1.000	Exact
Psyc Mgmt Antiaxiolytics	0 (0.0%)	157 (48.9%)	0 (0.0%)	25 (40.3%)	<0.001	Exact
Opioid Used >1	19 (19.0%)	63 (19.6%)	4 (23.5%)	16 (25.8%)	1.000	Exact

Trends identified provide a comparison point for local prevalence of marijuana use post state-wide legalization, which was found to be 15.8% (Table 2). Of the 79 with marijuana documentation, 13.9% of patients reported marijuana use to manage their anxiety (CI = 6.3%, 21.6%), and 17.7% reported use for pain management (CI = 9.3%, 26.1%). This study found a higher prevalence of cannabis usage in patients taking anti-psychotics (19.0%) compared to non-marijuana users (10.0%) with a p-value of 0.020 (Table 2). Additionally, it is important to note that most clinicians did not document a lack of marijuana use specifically in those considered a non-marijuana user (1.2%), but rather documented no illicit drug use in general.

## Discussion

An estimated 55 million American adults (16.9%) currently use marijuana in 2022 [20]. This number has increased when compared to a previous study in 2018 from the Annals of Internal Medicine which found that 14.6% of US adults reported using marijuana in the past year [21]. In the U.S., it is common to observe an increasing prevalence of marijuana use after legalization, but it is unknown if it is due to perceived risk or regional attitudes toward marijuana [2]. This increase has been observed in Colorado where adult marijuana use increased by 94% in the year 2019 since it was legalization [22]. With the increase in demand, use, and legalization approaching a national level, it is important to note the drastic increase in the prevalence of marijuana use.

This study provides insight into the local prevalence of marijuana use post-state-wide legalization (15.8%). As the data were abstracted at a time to coincide with the beginning of statewide legalization, this can serve as a comparison point for future analyses of prevalence in the state of Michigan, especially once the full implementation of the law occurred in 2019 following the passage of recreational marijuana legalization. It will be important from a public health perspective to continue researching trends in the prevalence of marijuana use moving forward.

This study also offers insight into the patterns of marijuana use in patients with a history of anxiety, depression, schizophrenia, those concomitant cocaine use, tobacco use, and psychotropic management. This study found that marijuana users were more likely to have a history of psychiatric disorder as well as current antipsychotic management. This further supports the association of active marijuana use with psychosis and mental health disorders such as schizophrenia, anxiety, and depression [4]. This association is important to recognize from a clinical perspective as physicians and practitioners screen for mental health disorders in patients with concurrent marijuana use, since these patients may be at higher risk than non-marijuana users, but further research is needed in this area. Additionally, there has not been sufficient clinical research to determine the safe amount of cannabis use to lower chronic pain and taper off opioids. One study did suggest that the legalization of marijuana may lead to a decrease in opioid overdose deaths [23], but further research is still needed for validation of the results.

Limitations

This study’s findings are subject to several limitations. Marijuana use screening is clinician and patient-dependent and thus, may be subject to reporting bias or clinician variability in screening methods. It is important to note that most clinicians did not document negative marijuana use in those considered a non-marijuana users (only 1.2% specifically documented non-marijuana use), but rather documented there was no illicit drug use in general. This may have led to the under-reporting of marijuana use since the legalization and increased accessibility of marijuana has led to greater societal acceptance that may have normalized marijuana as not being considered a drug (i.e., some patients may no longer consider it a drug or illicit substance). As marijuana undoubtedly continues to become more culturally acceptable secondary to its legalization and increase in access commercially, it will be critical for physicians and other healthcare professionals to query patients specifically on marijuana use in addition to illicit substance use when gathering a patient’s social history.

Furthermore, given the observational nature of this type of research study, correlations cannot determine whether there is a direct cause and effect relationship between marijuana use and other conditions. In addition, this study looked at only one healthcare system in one region in Michigan, therefore, the generalizability of these results may not apply to other regions in the State of Michigan or elsewhere.

## Conclusions

This study provides insight into the outcomes of the recent statewide legalization of recreational marijuana in mid-Michigan, as it pertains to patients being treated for conditions shown to be correlated with marijuana use. Because there is no previous data on marijuana use prevalence in the adult population in this region of Michigan, it is difficult to conclude how prevalence has changed in this population before and after the legalization of marijuana. As this data was abstracted at a time to coincide with the beginning of the statewide legalization, this can potentially serve as a comparison point for future analyses of prevalence in the state of Michigan. It will be important from a public health perspective to continue researching epidemiologic data and trends in the prevalence of marijuana use moving forward to inform public policy. Additionally, data at the state and local levels are important to help officials develop a more targeted prevention plan to ultimately prevent dependence, abuse, and adverse health effects associated with marijuana use.
